# Use of the Charge Transfer Reactions for the Spectrophotometric Determination of Risperidone in Pure and in Dosage Forms

**DOI:** 10.1155/2013/792186

**Published:** 2012-11-26

**Authors:** Hemavathi Nagaraju Deepakumari, Hosakere Doddarevanna Revanasiddappa

**Affiliations:** Department of Chemistry, University of Mysore, Manasagangothri, Mysore 570006, India

## Abstract

The aim of study was to develop and validate two simple, sensitive, and extraction-free spectrophotometric methods for the estimation of risperidone in both pure and pharmaceutical preparations. They are based on the charge transfer complexation reactions between risperidone (RSP) as *n*-electron donor and *p*-chloranilic acid (*p*-CA) in method A and 2,3-dichloro-5,6-dicyano-1,4-benzoquinone (DDQ) in method B as *π*-acceptors. In method A, RSP reacts with *p*-CA in methanol to produce a bright pink-colored chromogen measured at 530 nm whereas, in method B, RSP reacts with DDQ in dichloromethane to form orange-colored complex with a maximum absorption at 460 nm. Beer's law was obeyed in the concentration range of 0–25 and 0–50 *μ*g/mL with molar absorptivity of 1.29 × 10^4^ and 0.48 × 10^4^ L/moL/cm for RSP in methods A and B, respectively. The effects of variables such as reagents, time, and stability of the charge transfer complexes were investigated to optimize the procedures. The proposed methods have been successfully applied to the determination of RSP in pharmaceutical formulations. Results indicate that the methods are accurate, precise, and reproducible (relative standard deviation <2 %).

## 1. Introduction

**Figure 1:**
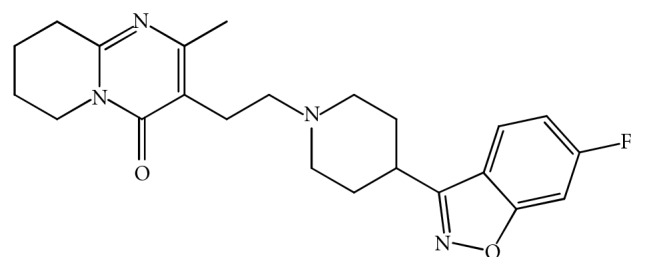
Structure of risperidone.

Risperidone(RSP)chemicallyknownas4-[2-[4-(6-fluorobenzo[*d*]isoxazole-3-yl)-1-piperidyl]ethyl]-3-methyl-2,6-diazabicyclo[4.4.0]deca-1,3-dien-5-one  (Figure  1), is the atypical antipsychotic drug with a relatively low incidence of extra pyramidal side effects. It is used for the treatment of schizophrenia, bipolar disorder, and behavior problems in people with autism. In 2003, the FDA-approved RSP for the short-term treatment of the mixed and manic states associated with bipolar disorder. It is also approved for the treatment of irritability in children and adolescents with autism in 2006. The drug is officially included in 2005 European Pharmacopeia, and the official method of its determination is high-performance liquid chromatography [1].

Many methods have been employed for the determination of RSP in biological samples including HPLC with electrochemical detection [2, 3] and RP-HPLC with UV detection [4]. The most extensively used technique for its determination is LC-MS/MS, but several procedures using this technique are confined to biological fluids like human plasma [5–8], plasma and urine [9], and serum [10]. A limited number of analytical methods for the quantitative estimation of RSP in pharmaceutical samples are known. Procedures based on high-performance liquid chromatography and thin-layer densitometric methods [11], spectrophotometry [12, 13], and gas chromatography [14] are available in the literature. The reported chromatographic techniques [11, 14] require expensive experimental setup, whereas the cited spectrophotometric methods: one is uv method [12] and another [13] requires extraction step for RSP determination. Thus, there is a need to develop sensitive, accurate, and economical methods for its determination. 

In the present study, the authors have described the development and validation of two simple and sensitive spectrophotometric methods for the analysis of RSP in pure form and in pharmaceutical samples using *p*-CA and DDQ as *π*-acceptors. The developed methods were validated for linearity, accuracy, and precision.

## 2. Experimental Section

### 2.1. Apparatus

All absorbance measurements were performed using a Systronics Model 166 digital spectrophotometer provided with 1-cm matched quartz cells.

### 2.2. Reagents and Standards

All chemicals and reagents used were of analytical reagent grade, and distilled water was used throughout the investigation.

(i) *p-Chloranilic acid* (0.05%, w/v): it was freshly prepared by dissolving 0.05 g *p*-chloranilic acid (Rolex, Mumbai, India) in 100 mL acetone.
(ii) *2,3-Dichloro-5,6-dicyano-1,4-benzoquinone* (0.1%, w/v): it was prepared by dissolving 0.1 g 2,3-dichloro-5,6-dicyano-1,4-benzoquinone (Avra synthesis Pvt. Ltd., Hyderabad, India) in 100 mL acetonitrile.
(iii) *Standard RSP solution*: pharmaceutical grade RSP, certified to be 99.98% pure, was received from Cipla India Ltd., Mumbai, India, as a gift sample and was used as such. A stock standard solution equivalent to 100 *μ*g/mL of RSP was prepared separately by dissolving 10 mg of the pure drug in 100 mL methanol in method A and in 100 mL dichloromethane in method B. Working solutions were prepared as required by dilution with respective solvents.

Pharmaceutical formulations of RSP such as Respidon (Torrent (Mind)) and Rispond (Micro Synapse) were purchased from local markets.

### 2.3. General Procedures for Calibration Graph

#### 2.3.1. Method A

An aliquot of standard solution containing 0.0, 0.25, 0.5, 1.0, 1.5,…, 5.0 mL (50 *μ*g/mL) of RSP was transferred into a series of 10 mL-calibrated flasks. To this solution was added 3.5 mL 0.05% *p*-CA, then shaken well, and the contents were diluted to the mark with methanol and mixed well. The absorbance of the bright pink-colored complex was measured at 530 nm after 5 min against the reagent blank prepared similarly, but without drug content. 

#### 2.3.2. Method B

Aliquots of a standard drug solution ranging 0.0, 0.5, 1.0, 1.5, 2.0 …, 5.0 mL (100 *μ*g/mL) were taken in a series of 10 mL-calibrated flasks. Then, to each flask 1.5 mL of 0.1% DDQ was added. The contents were diluted to the mark with dichloromethane and mixed well, and the absorbance of the colored product was measured at 460 nm against the reagent blank. The amount of RSP present in the sample was computed from calibration curve or the regression equation.

### 2.4. Procedure for Pharmaceutical Preparations

Thirty tablets each containing 1 mg of RSP were weighed and finely powdered. An accurately weighed amount of the powder equivalent to 10 mg of RSP was transferred separately into 100 mL-calibrated flasks and 10 mL each of methanol for method A and dichloromethane for method B was added. The content was shaken for about 30 min; the volume was diluted to the mark with respective solvents and mixed well and filtered using a Whatman No. 41 filter paper. The filtrate containing RSP (at a concentration of 100 *μ*g/mL) was subjected to analysis by the procedures described above.

### 2.5. Procedure for the Analysis of Placebo Blank and Synthetic Mixture

A placebo blank containing starch (10 mg), acacia (15 mg), hydroxyl cellulose (10 mg), sodium citrate (5 mg), talc (15 mg), magnesium stearate (20 mg), and sodium alginate (10 mg) was prepared by combining all components to form a homogeneous mixture, and its solution was prepared as described under “Procedure for pharmaceutical preparations” and was subjected to analysis by following the general procedures. A synthetic mixture was separately prepared by adding pure RSP (20 mg) to placebo blank, and the extract was prepared by diluting to give RSP (100 *μ*g/mL) and was used in both methods A and B, respectively.

 Synthetic mixture solution prepared above was taken at three different concentrations equivalent to 5, 10, and 15 *μ*g/mL in method A and 10, 20, and 30 *μ*g/mL in method B and was subjected to analysis by following the general procedures. The results of the study indicate that the common tablet excipients did not interfere in the assay.

### 2.6. Stoichiometry

**Figure 2:**
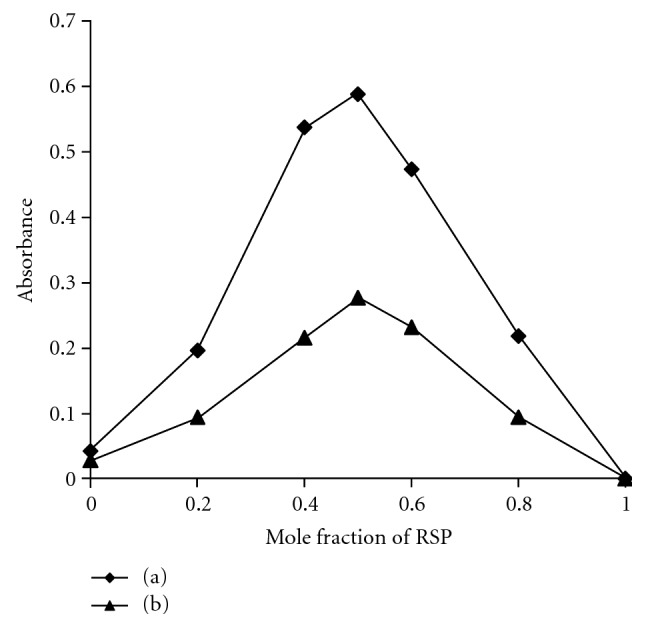
Job's plot for stoichiometric ratio for (a) (RSP) and (*p*-CA) = (1.22 × 10^−4^ M) and (b) (RSP) and (DDQ) = (2.44 × 10^−4^ M).

Job's method of continuous variation [15] was employed to establish the stoichiometry of the colored products. The solutions equivalent to 1.22 × 10^−4^ and 2.44 × 10^−4^ M RSP were prepared. Further, 1.22 × 10^−4^ M *p*-CA and 2.44 × 10^−4^ M DDQ solutions were prepared in acetone and acetonitrile, respectively. A series of solutions were mixed in complimentary proportions; in method A, the volume was completed up to the mark using methanol, and with dichloromethane in method B. The absorbances of the resulting solutions were measured at their respective wavelengths (*λ*_max_) against the reagent blank under the similar conditions. Job's method of continuous variations graph for the reaction between RSP and *p-*CA or DDQ (Figure 2) shows that the interaction occurs on an equimolar basis *via *the formation of charge-transfer complexes in the ratio 1 : 1 (RSP : *p-*CA or DDQ).

## 3. Results and Discussion

### 3.1. Chemistry of the Colored Product

The methods involve charge-transfer (C-T) complex formation between the basic nitrogenous RSP as *n*-donor and *p*-chloranilic acid (*p*-CA) and DDQ as *π*-acceptors in polar solvents. In each case, the formed charge-transfer complex was subsequently dissociated into radical anions, which are colored species. In method A, an intense bright-pink-colored product was formed by the interaction of donor *n*-electrons of RSP, and *π*-acceptor *p*-CA in acetone-methanol solvent system showed absorption maxima at 530 nm due to the formation of the corresponding *p*-CA radical anion. A DDQ-RSP charge transfer complex exhibits a maximum absorption at 460 nm; this is due to the formation of DDQ radical anion arising from the complete transfer of *n*-electrons from RSP to acceptor DDQ in acetonitrile-dichloromethane solvent, in method B.

In polar solvents such as methanol or dichloromethane, complete electron transfer from the donor to the acceptor moiety takes place with the formation of intensely colored radical anions [16], as per the following equation:

(1) $$\begin{array}{c} \ddot{\text{D}}+\text{A}\to \underset{\text{CT complex}}{\left[\ddot{\text{D}}\to \text{A}\right]}\overset{\text{Polar Solvent}}{\to }{\dot{\text{D}}}^{+}+\underset{\text{Colored radical anion}}{{\dot{\text{A}}}^{-}}. \end{array}$$

**Scheme 1:**
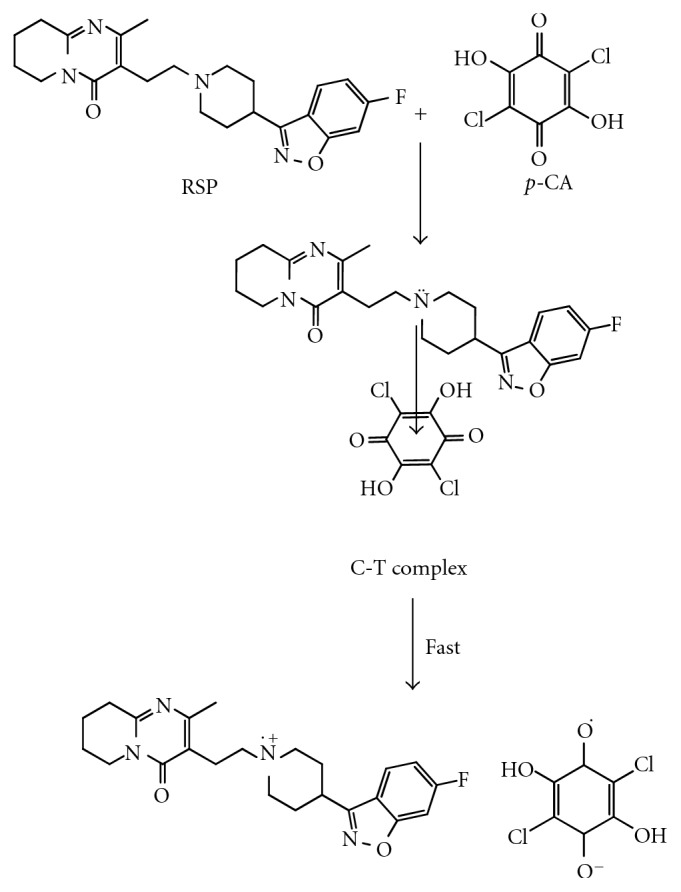
Proposed reaction scheme for method A.

**Scheme 2:**
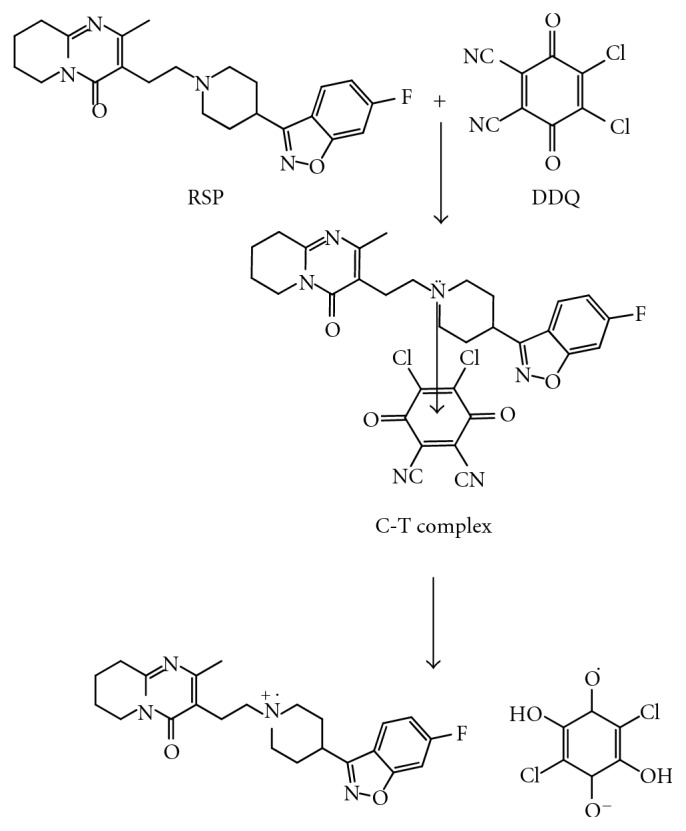
Proposed reaction scheme for method B.

Thus, *p*-CA and DDQ were used as reagents in the proposed methods A and B, respectively, for the estimation of RSP. The possible reaction pathway for RSP-*p*-CA and RSP-DDQ complexes was proposed and depicted in Schemes 1 and 2, respectively.

The reaction stoichiometry between RSP and *p*-CAor DDQ was evaluated by applying Job's method of continuous variations. Job's plot (Figure 2) reacheda maximum value at a mole fraction of 0.5 which suggested a donor (RSP) to acceptor (*p*-CA or DDQ) ratio of 1 : 1. This indicated the presence of *n*-donating center in the RSP base for charge transfer complexation reaction. 

### 3.2. Optimization of Experimental Parameters

The factors affecting the formation of charge-transfer complexation, reproducibility, sensitivity, and adherence to Beer's law were investigated and are reported below. 

#### 3.2.1. Effect of *p*-CA Concentration

In order to study the effect of the volume of the reagent on the absorbance of the charge transfer complex, varying volumes of 0.05% *p*-CA were mixed with 10 *μ*g/mL drug in a 10 mL-calibrated flask and diluted to volume with methanol. Highest absorbance was obtained with 3.0 mL, which remained unaffected by further addition of *p*-CA. Hence, 3.5 mL of the reagent was used for the determination of RSP in method A.

#### 3.2.2. Effect of DDQ Concentration

To establish the optimum experimental condition, risperidone (10 *μ*g/mL) was allowed to reactwith different volumes (0–3 mL) of 0.1% DDQ. Highest absorbance was obtained with 1.0 mL, which remained unaffected by further addition of DDQ. Thus, a volume of 1.5 mL of DDQ was used for the determination of RSP in method B.

#### 3.2.3. Effect of Time and Stability of the Complex

The optimum reaction time was evaluated by monitoring the color development upon the addition of reagent solution to RSP at roomtemperature. It was observed that the reaction got stabilized within 5 and 2 min in methods A and B, respectively. The developed colorwas remained stable for 60 min at room temperature for both the methods. 

### 3.3. Method Validation

According to the ICH guidelines [17], both the methods were validated for linearity and sensitivity, limit of detection (LOD) and limit of quantitation (LOQ), precision, accuracy, selectivity, and recovery.

#### 3.3.1. Linearity, Sensitivity, Limits of Detection, and Quantification

To establish the linearity, accuracy, and precision under optimized experimental conditions for both methods A and B. A linear correlation was found between the absorbance at respective wavelengths, and concentrations of RSP in the ranges are given in Table 1. Regression analysis of the calibration curve using the method of least squares was made to calculate the slope (*b*), intercept (*a*), and correlation coefficient (*r*) for each method (methods A and B), and the values are presented in Table 1. The optical characteristics such as absorption maxima, Beer's law limit, molar absorptivity, and Sandell's sensitivity values of two methods are also given in Table 1. 

The limit of detection (LOD) and limit of quantitation (LOQ) were evaluated as per ICH guidelines using the following equations:

(2) $$\begin{array}{c} \text{LOD}=\frac{3.3\times \sigma }{s},\\ \text{LOQ}=\frac{10\times \sigma }{s}, \end{array}$$

where *σ* is the standard deviation (*n* = 5) of reagent blank determination, and *s* is the slope of the calibration curve.

#### 3.3.2. Precision and Accuracy

The precision and accuracy (intra-day and inter-day) of the methods developed were evaluated by replicate analysis of drug samples at three different concentrations (low, medium, and high) (Table 2) within the working limits, each being repeated five times. The RE (%) and RSD (%) values of both intra and inter-day studies were less than 2.0 and showed the best appraisal of the procedures in daily use:

(3) $$\begin{array}{c} \text{RE}\%\;=\left[\frac{\text{founded}-\text{added}}{\text{added}}\right]\times 100. \end{array}$$

The analytical results obtained from this investigation are summarized in Table 2. The values of percentage relative error between the concentrations of RSP for taken and found showed the high accuracy of the methods. The results obtained are presented in Table 2 and showed that the accuracy is good.

#### 3.3.3. Application to Analysis of Pharmaceutical Samples

To check the validity of the proposed charge-transfer spectrophotometric methods, RSP was determined in some commercial formulations. The result obtained from the determination is in close agreement between the results obtained by the proposed methods and the label claim. Statistical analysis of the results using Student's *t*-test for accuracy and F-test for precision revealed no significant difference between the proposed methods and the literature method [12] at the 95% confidence level with respect to accuracy and precision (Table 3).

#### 3.3.4. Recovery Study by Standard Addition Technique

The accuracy and precision of the proposed methods for the determination of RSP in commercial sample were further ascertained by performing recovery studies. In this study, preanalyzed tablet powder was spiked with pure drug at three different concentrations, and the total was found by the proposed methods. Each determination was repeated three times. The recovery of the pure drug added was quantitative and revealed that coformulated substances did not interfere in the determination. The results of recovery study are compiled in Table 4. 

## 4. Conclusions

The present paper describes two simple, accurate, precise, and sensitive extraction-free spectrophotometric methods for the determination of risperidone in bulk drug and in tablet. The methods rely on the use of simple and cost-effective chemicals in both the methods and can be successfully applied to the routine estimation of risperidone in bulk and tablet dosage forms. From the calculated *t*- and *F* values at the 95% confidence level, it is clear that the results obtained by the proposed methods are in good agreement with those obtained by the reference method [12]. The small values of RE and RSD indicate the reliability, accuracy, and precision of suggested procedures. The results obtained in Tables 3 and 4 are considered to be of high accuracy, and, therefore, these methods can be recommended for the routine analysis of risperidone in quality control laboratories. 

## Figures and Tables

**Table 1:** Analytical and regression parameters of the proposed methods.

Parameter	Method A	Method B
*λ*_ max_ nm	530	460
Beer's law range (*μ*g/mL )	0–25	0–50
Molar absorptivity (*ε*), (L mol/cm)	1.29 × 10^ 4^	0.48 × 10^ 4^
Sandell's sensitivity (*μ*g/cm^2^)	0.0319	0.0852
Regression equation∗		
Intercept (*a*)	0.0152	0.0097
Slope (*b*)	0.0296	0.0104
Correlation coefficient (*r*)	0.997	0.997
*S*_*a*_	0.0325	0.0216
*S*_*b*_	0.0014	0.0005
LOQ (*μ*g/mL)	0.6521	1.6884
LOD (*μ*g/mL)	0.2152	0.5572

^*^ *y* = *a* + *bx*, where *c* is the concentration of RSP in *μ*g/mL, *y* is the absorbance at therespective *λ*_max_, *S*_*a*_ is the standard deviation of the intercept, and *S*_*b*_ is the standard deviation of the slope.

**Table 2:** Evaluation of accuracy and precision.

Method	RSP taken, *μ*g/mL	Intra-day accuracy and precision			Inter-day accuracy and precision		
		RSP found∗, *μ*g/mL	% RE	% RSD	RSP found, *μ*g/mL	% RE	% RSD
Method A	5	4.98	0.43	0.41	5.03	−0.49	0.23
	15	14.89	0.74	0.39	15.05	−0.33	0.24
	20	19.93	0.35	0.28	20.14	−0.67	0.39

Method B	5	4.98	0.42	0.79	5.11	−2.28	0.79
	20	19.78	0.94	0.35	20.03	−0.12	0.39
	40	39.89	0.28	0.27	40.12	−0.29	0.53

RE: relative error; RSD: relative standard deviation.

∗Mean value of 5 determinations.

At the 95% confidence level for 4 degrees of freedom.

**Table 3:** Results of determination of RSP in tablets and statistical comparison with the reference method.

Tablet brand name	Nominal amount mg per tablet	Found∗∗ (% of nominal amount ± SD)		
		Reference method [12]	Method A	Method B
Respidon^a^	1 mg	102.0 ± 0.18	100.18 ± 0.14	100.92 ± 0.45
			*t* = 0.87, *F* = 1.73	*t* = 2.68, *F* = 6.23

Rispond^b^	1 mg	101.8 ± 0.24	99.28 ± 0.19	101.16 ± 0.45
			*t* = 1.04, *F* = 0.66	*t* = 1.45, *F* = 3.57

Marketed by: ^a^(torrent (mind)), ^b^(micro synapse); ∗∗mean value of five determinations.

Tabulated *t* and *F* values at 95% confidence level are 2.77 and 6.39, respectively.

**Table 4:** Results of recovery experiments via the standard addition technique.

Tablet brand name	Method A				Method B			
	RSP tablet *μ*g/mL	Pure RSP added, *μ*g/mL	Total found *μ*g/mL	Pure RSP recovered∗ % ± SD	RSP tablet *μ*g/mL	Pure RSP added, *μ*g/mL	Total found *μ*g/mL	Pure RSP recovered∗ % ± SD
Respidone (torrent (mind))	5	5	9.97	99.22 ± 0.19	10	10	19.99	99.86 ± 0.48
	5	10	15.10	100.91 ± 0.11	10	20	30.25	101.23 ± 0.37
	5	15	20.08	100.42 ± 0.13	10	30	40.50	101.67 ± 0.50

Rispond (micro synapse)	5	5	9.92	98.38 ± 0.16	10	10	20.02	100.19 ± 0.27
	5	10	15.02	100.16 ± 0.23	10	20	30.27	101.35 ± 0.95
	5	15	19.89	99.28 ± 0.19	10	30	40.59	101.96 ± 0.14

∗Mean value of three measurements.
